# A nationwide Chinese consumer study of public interest on agriculture

**DOI:** 10.1038/s41538-022-00147-1

**Published:** 2022-07-07

**Authors:** Danfeng Liao, Kai Cui, Lijing Ke

**Affiliations:** 1 Science popularization Office, China Association of Agricultural Science Societies, 100125 Beijing, China; 2 Antai College of Economics and Management (ACEM), Shanghai Jiao Tong University, 200030 Shanghai, China; 3 The School of Food Science and Biotechnology, Zhejiang Gongshang University, 310065 Zhejiang, China

**Keywords:** Agriculture, Science, technology and society, Education

## Abstract

A nationwide study was undertaken in China to understand why public interest has shifted away from agriculture and to discuss approaches that may help restore interest and support for agriculture. The study collected 2586 questionnaires from 242 cities in 31 provinces in mainland China. The results suggest that agriculture is still of public interest, but interest has shifted from traditional farming to the consumer perspective in food safety, nutrition and health, food security and agricultural history. Two groups in this study, the younger generation and those with college degrees, show less interest in production agriculture. The accelerating shift in population from rural China to urban areas explains why these two groups are less connected with agricultural issues. The authors contend that it is critically important to keep the urban population knowledgeable of the importance of agriculture and suggest ways to improve communication and support from this educated, city-dweller point of view in order to ensure a stable and secure future. The approach of science appreciation (ways to effectively communicate science to general publics) is proposed to effectively gain renewed interest and engagement with the public in the science of agriculture in order to optimize the needs and benefits from agriculture to society.

## Introduction

Since the 1980s, industrialization has transformed China’s agricultural landscape. The classic scene of “harvesting grains and livestock husbandry” in the Chinese agricultural tradition is gradually fading away. Agriculture has been transformed from a leading sector in the nation’s economy (30% of GDP, 1980) to an average one (8% of GDP, 2020). Meanwhile, China’s urbanization population has increased from 20% in 1980 to 60% in 2019 and is expected to further increase to 70% and 80% in 2030 and 2050, respectively^1^.

Increasing urbanization has created a gap in the appreciation of consumers and modern agriculture practices and how food is produced. Issues like food safety, nutrition and health, animal welfare and the environmental impact of modern agriculture should be of major concern to society. The World Health Organization (WHO) advocates a One Health approach where the health of citizens, the health of animals and the health of the environment are inextricably linked (https://www.who.int/news-room/questions-and-answers/item/one-health). China is no different than other countries where citizens are becoming less aware of the importance of agriculture and food production for sustainability.

The agri-food sector has never been more important and to ensure food security it will require public understanding and support. The authors therefore undertook a nationwide study to gather data on the public’s current general attitude and understanding of agriculture and how to use this information to optimize the approach of science appreciation and engagement with the public on issues relating to agriculture. Greater public understanding and support for the critical need of a robust agricultural sector to ensure future food and health security for all its citizens is required.

The study focused on surveying a large cross-section of people from China on 24 agriculture-related topics. The study included 2586 participants, covering 242 cities in 31 provinces in mainland China. The overall demographic characteristics of the surveyed participants, including their location, their age, educational background, and gender are summarized in Table 1 according to overall percent in each category. The data were analyzed by SPSS software. The Cronbach’s *α* was 0.912 which illustrated the survey was highly reliable in content consistency.

**Table 1:** Demographic characteristics of the surveys.

Class	Sub-class	Percentage	Class	Sub-class	Percentage
Region	4 Municipalities	24.2	Age	Under 25	25.0
	9 provinces in Eastern China	33.1		26-50	61.6
	9 provinces in Central China	34.0		Over 50	13.4
	9 provinces in Western China	8.6	Education	High school and lower	13.0
Gender	Male	52.9		University/College	59.1
	Female	47.1		Postgraduate	27.8

## Results

The male and female ratio of the respondents was about 53% and 47%, respectively, which indicates a balanced gender distribution. The respondents were dominated by the age group of 26–50 years old, accounting for 61.6%. The education level of respondents was mainly university and above, while the university graduates accounted for 59.1% and postgraduates accounted for 27.8%.

### Differences in public interests

Among the 25 questions asked to the participants, one two-part question served as a control for this study to calibrate the level of interest. It is related to the hot topic of the COVID pandemic and the COVID-19 vaccine. Participants were asked, “How effective is a COVID-19 vaccine?” and “When will life return to normal as it was in the pre-pandemic era?”. Their interests to these two questions were recorded regardless the attitude or judgment they might hold. The 25 questions randomly asked in the questionnaire are listed in Table 2 according to the percentage of people showing interests, from high to low. The responses were used as a measure of the public’s level of interest to agricultural topics (Q1–Q24, Table 2). Not surprisingly, the level of interest to the COVID-19 related control questions was the highest at 55.6%. There were five agricultural topics attracting interests of over 40% of the participants. Among the 24 agricultural questions, the highest percentage was 48.6%, while the lowest one was 21.0%.

**Table 2:** Questions ranked according to public interest.

Sequence number	Questions	Interest (%)
Control	How effective is the COVID-19 vaccine? When will the life return to normal as it was in the pre-pandemic era?	55.6
Q01	Why is bread favored in the West, while steamed buns in China?	48.6
Q02	Why do fruits and vegetables taste not as good as they used to?	47.5
Q03	Why are grains, instead of fruits or meat, our staple food?	46.4
Q04	Which crops are genetically modified? Is genetically modified food safe?	43.2
Q05	What are the major famines in human history and their main causes?	40.6
Q06	What is the difference between craft beer and regular beer? What’s cultivated or plant-based meat?	38.3
Q07	What are the influences of fertilizers, pesticides, additives, and hormones on the environment and food safety?	38.1
Q08	What is the difference between ancient and modern food?	38.1
Q09	Which food could assist weight loss? Does a vegetarian diet affect health?	37.7
Q10	Does China have food security challenges? How to solve the problem of food waste?	37.4
Q11	With the growth in agricultural production, why do farmers still have a difficult time?	36.4
Q12	Why can the United States become the leading country in agriculture? Why does China import massive number of soybeans from the States?	35.7
Q13	How did China become an agricultural power? What will Smart Agriculture be like?	35.3
Q14	China’s population is aging. Will the lack of labor affect agricultural production in the future?	35.0
Q15	Farmers used to keep their own seeds. However, diversity and yield of commercial seeds will be reduced if they are kept under private control. Why?	34.8
Q16	What are the influences of global warming on agriculture?	34.4
Q17	How were weeds domesticated into grains in ancient times?	32.4
Q18	How to keep agricultural culture heritage sites and traditional rural practices?	31.9
Q19	Where did the world’s first farmer emerge?	29.1
Q20	Does rice originate in China or India?	28.0
Q21	What are impacts on agriculture if biodiversity is compromised?	27.5
Q22	What are the planting processes of crops (grain, vegetables, fruits)?	24.3
Q23	How to produce biofuel (i.e. ethanol from corn)? Does it mean that cars are competing with people for food?	23.0
Q24	What are the farming practices for livestock and poultry (cattle, horses, pigs, chickens)?	21.0

The above 25 questions include control questions and 24 agricultural-related questions. They are referred in the main text by their sequence number as labeled above.

In general, the five questions with more than 40% interest rate emphasized life, history, and cultural perspectives, while the six questions with interest rates <30% were mainly in the field of agricultural science. The interest rates of the other 13 questions were between 30% and 40%, mainly covering food security, food safety, nutrition and health, emerging food, agricultural development, etc. These findings support changes in the participants’ dietary habits and structure, from “filling” to “eat well” to “healthy eating” over the past 40 years, with new thoughts in agriculture.

There are several sets of data worthy of interest when promoting science appreciation. For example, the interest percentages of Q2 (Why do fruits and vegetables taste not as good as they used to?) and Q4 (Which crops are genetically modified? Is genetically modified food safe?) were 47.5% and 43.2%, respectively. To fully understand the responses to these questions, it is necessary to understand Q22 (What are the planting practices of crops?) and Q24 (What are the farming practices of livestock and poultry?). However, the interest percentages of Q22 and Q24 was only 24.3% and 21%, respectively. The ranking of these two questions in different groups were mostly 22nd/23rd and 24th. This result supports the view that the public tends to “care about the result, but not how it was achieved”.

The respondents were interested in emerging foods, such as craft beer and cultivated/plant-based meat (Q6), agricultural product safety (Q7), food security (Q10), and increasing farmers’ income (Q11). In contrast, interest to global warming (Q16), biodiversity (Q21) and inheritance and conservation of rural civilization (Q18) was not high. These are major issues for society and the low level of interest implies that there is a need to improve and strengthen the related science appreciation efforts to increase public awareness and understanding.

### Demographic analysis of interest level

Public attitudes and opinions tend to be diversified in the era of the internet^2^ and our survey findings demonstrate that this is the case with agricultural topics. The interest level (percentage) was analyzed and presented in groups according to age, education, and gender of the participants (Table 3).

**Table 3:** Demographic analysis of interest percentage.

Seq. no.	Age			Education			Gender	
	Under 25	26–50	Above 50	High school and lower	University/College	Post-graduate	Male	Female
Q01	45.0	48.9	53.7	49.6	46.4	52.9	47.1	50.3
Q02	**38.2**	**49.0**	**58.0**	50.4	47.4	46.4	**42.6**	**53.0**
Q03	43.5	45.1	57.8	49.6	45.5	46.8	44.7	48.4
Q04	**26.2**	**46.9**	**57.8**	44.5	42.2	44.6	40.4	46.3
Q05	35.8	40.6	50.0	48.4	38.5	41.5	42.8	38.3
Q06	38.1	38.1	39.7	36.5	38.5	38.9	38.2	38.4
Q07	**23.8**	**39.4**	**58.3**	48.1	37.5	34.7	38.6	37.5
Q08	38.7	36.9	42.2	41.2	37.1	38.5	38.3	37.8
Q09	37.8	37.4	39.1	36.8	37.3	39.2	**28.9**	**47.7**
Q10	**26.2**	**37.5**	**58.0**	41.2	35.7	39.3	36.8	38.2
Q11	30.3	36.4	47.7	**49.6**	**35.9**	**31.3**	37.6	35.1
Q12	**28.3**	**35.8**	**49.1**	34.4	34.3	39.3	35.4	36.1
Q13	**26.2**	**35.1**	**50.9**	40.4	35.3	31.8	34.9	35.0
Q14	27.7	36.0	46.6	37.4	34.5	36.1	36.4	34.2
Q15	**27.4**	**34.7**	**49.1**	46.3	34.4	30.3	33.9	35.8
Q16	29.4	33.7	46.6	43.0	34.8	29.4	35.7	32.9
Q17	38.7	29.6	33.6	32.0	33.3	30.7	34.9	29.6
Q18	30.8	30.8	38.5	35.0	33.1	27.8	31.0	32.8
Q19	31.6	27.0	34.2	**43.6**	**29.0**	**22.6**	32.5	25.4
Q20	24.6	26.8	40.2	**41.5**	**28.5**	**20.7**	31.1	24.5
Q21	23.5	26.2	40.5	31.8	26.8	26.8	28.3	26.5
Q22	22.9	23.9	28.7	**36.2**	**24.3**	**18.9**	26.1	22.3
Q23	20.7	22.6	29.0	32.0	22.7	19.4	25.1	20.6
Q24	19.8	20.2	27.3	30.6	20.9	16.8	24.0	17.7
Mean	30.6	34.9	44.9	40.8	34.7	33.5	35.2	35.2

The data are labeled in bold font if the percentage difference among groups is >20%.

Many people over the age of 50 have lived in rural areas while the younger generation who grew up in cities lack the experience of rural life. According to the data in Table 3, the older the participant is, the higher their interest is to the questions. On average, it was 30.6% for the group of 25 years old and younger, 34.9% for the group of 26–50 years old and 44.9% for people in their 50s and older. Among the 24 questions, 23 were deemed more important to those over 50 years old than those 25 years old and younger. The exception was Q17 (How were weeds domesticated into grains in ancient times?). In this case, young participants were more interested. There were 7 questions (Table 3, shown in bold font) whose difference of its interest percentage among different age groups was >20%.

In terms of educational qualifications, it was observed that respondents with higher qualifications had less interest in questions on agriculture. The average percentage found for each group was 40.8% (high school and lower), 34.7% (university/college), and 33.5% (postgraduate), separately. Nineteen of the questions were of higher interest to the group with high school and a lower level of education, while the other five questions were of more interest to those who had postgraduate training. There were 4 questions (Q11, Q19, Q20, Q22) whose interest percentage difference between these two groups was around 20% or greater (Table 3, shown in bold font).

There were no significant differences found between genders. The average interest percentages in both men and women were 35.2%. Among the 24 questions, 22 questions attracted similar level of interest from both groups. However, men showed much less interest than their female counterparts in two questions: Q02 (Why do fruits and vegetables taste not as good as they used to?) and Q09 (Which food could assist weight loss? Does a vegetarian diet affect health?).

### Demographic analysis of interest ranking

In addition to the above analyses, some additional data are worth noting. For example, the interest percentage of different age groups was generally very close to each other for Q06 (What is the difference between craft beer and regular beer? What’s cultivated or plant-based meat?). It was 38.1% (under 25), 38.1% (26–50), and 39.7% (over 50). However, the ranking of this question in each of the age groups according to their interest percentages was rather different, which was 6th, 7th, and 17th, respectively. To address these differences, the interest ranking of the 24 questions was further analyzed (Table 4).

**Table 4:** Demographic analysis of interest ranking.

Seq. no.	Age			Education			Gender	
	Under 25	26–50	Above 50	High school and lower	University/College	Postgraduate	Male	Female
Q01	1	2	6	2	2	1	1	2
Q02	5	1	2	1	1	3	4	1
Q03	2	4	4	3	3	2	2	3
Q04	***18***	***3***	***5***	8	4	4	5	5
Q05	8	5	8	6	5	5	3	7
Q06	6	7	17	17	6	9	8	6
Q07	4	10	1	12	9	10	7	9
Q08	20	6	14	5	7	12	6	10
Q09	7	9	18	16	8	8	***20***	***4***
Q10	***16***	***8***	***3***	13	11	6	10	8
Q11	11	11	11	4	10	14	9	13
Q12	13	13	10	***20***	***16***	***7***	13	12
Q13	14	12	12	15	14	11	11	15
Q14	17	14	7	14	12	13	14	14
Q15	15	15	9	7	15	16	16	12
Q16	12	16	13	10	13	17	12	16
Q17	***3***	***18***	***21***	21	17	15	15	18
Q18	10	17	19	19	18	18	19	17
Q19	9	19	20	9	19	20	17	20
Q20	19	20	16	11	20	21	18	21
Q21	21	21	15	23	21	19	21	19
Q22	22	22	23	18	22	23	22	22
Q23	23	23	22	22	23	22	23	23
Q24	24	24	24	24	24	24	24	24

1, highest rank; 24, lowest rank. The data are labeled in italics and bold font if the ranking difference among different groups is >12.

The results showed that 19 questions shared a consistent level of interest (percentage versus ranking), particularly the top three questions and the last three questions. In terms of age groups, there were three questions whose differences in ranking were more than 12 (half of the 24 questions). In terms of educational qualifications and gender, there was only one question whose ranking difference among different groups was >12.

In terms of age, Q04 (Which crops are genetically modified? Is genetically modified food safe?) ranked 18th among participants under 25 years old, 3rd for those age 26–50 and 5th for those over 50 years old. In other words, “post-95” young people do not seem to be concerned about the debate on genetically modified crops and foods. This is consistent with previous reports^3^. Another example is Q10 (Does China have food security challenges? How to solve the problem of food waste?). Although food security includes issue of food waste, it would make better sense for participants to understand the context of question by presenting these two questions together. This question ranked the 16th among participants under 25, 8th among those age 26–50 and 3rd among those over 50 years old. It seems to echo a saying “those who are not in charge of daily expenses have no idea how expensive it could be”. The youngest group showed much higher interest in the questions like Q17 (How were weeds domesticated into grains in ancient times?) compared to older groups. This question ranked 3rd among those under 25 years old. The same question ranked only 18th and 21st when asked to participants aged 26–50 years old and those over 50 years old, respectively.

In terms of educational qualifications, the higher the education the higher the rank for questions like Q12 (Why can the United States become the leading country in agriculture? Why does China import massive number of soybeans from the States?). The response was 20th for the “high school and lower”, 16th for university/college graduates, and the 7th for those who hold post-graduate level degrees. This difference can be correlated to the aspiration and vision of people with more education. In terms of gender, the health-related question Q09 (Which food could assist weight loss? Does a vegetarian diet affect health?) ranked much lower (20th) among men compared to women (4th).

## Discussion

In recent years, the old-school “science appreciation” books have been of little or no value for many publishing houses^4^, and subscriptions to journals of agricultural science have declined sharply^5^. It has become a common phenomenon for students at agricultural colleges and universities to be “learning agriculture, hating agriculture, and abandoning agriculture”^6^. Despite the complicated reasons behind this trend, it is worth rethinking how agricultural topics are communicated in classrooms and to the public. Changing the presentation style to enhance interest and engagement can be achieved by incorporating stories about popular aspects of agriculture combined with good depth and breadth of comprehensible knowledge. Based on the results of this survey we suggest four approaches to improve communicating topics related to agricultural science.

First, general knowledge must be taught at school or via multiple educational platforms. Urban civilization is embodied in history, science and technology, and humanistic care. Such characteristics were reflected in this study. The public has shown more interest in food culture, origins of food, and famine, while less interest to pure scientific topics. In order to understand the above topics of interest, it will require a certain level of general knowledge as a foundation to build further knowledge and create a greater understanding of agriculture and where food comes from.

Agriculture is a broad and deep field with many topics attracting public interest. The implementation of agricultural science communication requires innovation in the body of knowledge and the way of thinking about it. For example, research on the origin of grains not only allows biologists to explore genetic resources of wild species and improve crop varieties, but also helps anthropologists build ethnic history. Food security requires consideration of population, resources, and environment as well as the global trading system. The development of bioenergy has connected corn, sugar cane, soybeans, and petroleum, which at one time seemed irrelevant, to a single supply chain with each piece relating to another. The debate over genetically modified organisms involves many topics including the public’s right to know, bioethics, environmental protection, government supervision, media and communication, commercial profits, international trade, and national sentiments^3,7^.

Second, the awareness of crisis. Communication of agricultural science should enable the public to truly understand the crises and challenges faced by the nation’s agriculture. To boost the yield of crops, chemical fertilizers and pesticides have been used in excessive quantities, subsequently caused many environmental problems such as soil compaction and water contamination^3,8^. Seeds are the “core” of agriculture. China’s seed production is generally lacking international competitiveness. According to this survey, only 37.4% of the public are concerned about food security and food waste (Q10), and 34.8% of the public are concerned about seeds (Q16). Both questions shared a rather low interest ranking. China is the world’s most populated country and the number one food importer. The 120 million tons of agricultural products imported annually is equivalent to 53 million hectares of arable land, which is equivalent to 40% of China’s 133 million hectares of arable land. Over reliance on imports to feed the nation is not a satisfactory situation so the development of the agri-food sector should be of concern to every citizen in China.

Starting from 1962, it took South Korea 30 years to complete industrialization. The urban population increased from 28% in 1960 to 74% in 1990, making it one of the “The Four Little Dragons”. However, along with the economic success, the position of agriculture in South Korea has been significantly weakened. People neglected the fact that the poor rate of food self-sufficiency, as reflected in significant food waste^9,10^. It is not difficult to hypothesize that China will have a similar problem to that of Korea. The lesson should be learned as otherwise China will experience weaknesses in their agri-food production systems.

Third, agriculture needs to be close to life. The public has changed from agricultural producers to today’s food consumers, whose interest to agriculture has transformed from “farm” to “taste”. The food chain connects the farm, where raw foods are initially produced, all the way to dining tables, providing food to fulfill the various needs of modern customers. The above results show that topics related to the consumption of food received high attention, while the production of food was falling out of the public’s general interest. If we are to have a sustainable society, we need sustainable food production systems and we need greater public awareness and interest. This may be achieved if we increase our efforts to popularize agricultural science and food production covering the entire food chain from farm to fork.

Forth, humanistic care should be promoted. The progress of industrialization is inevitably accompanied by the fading away of rural practices, old cultures, pastoral songs and poems, etc., which are now hardly remembered by anyone. Farming and advances in agriculture were the foundation for progress and industrial development. However, even tens of thousands of years later, the root of humanity would still rely on the fertile land with its grains and crops. It is the authors’ hope that modern agriculture can reposition farming and food production so that it is recognized as central to the nation’s development. The national spirit accumulated from the thousands of years of China’s farming civilization can be rekindled and will nurture us towards a brighter and sustainable future. This is a China-centric perspective but also could be a global perspective.

## Methods

### Survey design

To identify the key topics in agriculture, we summarized more than 5000 questions and comments from the audience and readers from several popular science lectures and Wechat posts from 2016 to 2020 in China. The authors interviewed more than 100 scholars, journalists, officials and business managers who are interested in agriculture, and collectively selected 24 main issues of public concern.

These issues were classified to 4 areas:

(1) agricultural science;

(2) food safety and environmental protection;

(3) food security and national policy;

(4) history and anthropology.

An initial question on the COVID-19 pandemic was used as the calibration for the subsequent 24 questions relating to agriculture. From December 1 to 10 in 2020, this study selected 100 respondents in Shanghai City and Shandong Province to conduct on-site trial surveys. The respondents came from various disciplines of study and had no direct link to agri-food sector to reduce the chance of bias. They represent the better educated population in cities. Based on the feedback, the expression and wording of the questionnaire was revised and improved, and presented as the formal questionnaire for the further investigation.

### Investigation process

This survey was carried out on a mobile platform of questionnaire called Wen-Juan-Xing during December 21–28, 2020, covering age group of 18 years old and above. A total of 2586 valid questionnaires were recovered from residents in 242 cities located in 31 provinces, municipalities, and autonomous regions in Mainland China. The questions were presented in a random order. The participants voluntarily participated in the survey and were asked to pick the questions that interest them. Ethical review and approval was not required for the study, nor was written informed consent from the survey respondents to participate in this study, in accordance with national legislation and institutional requirements. The level of interest was measured by calculating the percentage of respondents who were interested in each question. The data were analyzed by SPSS software.

## Data Availability

The authors declare that all data supporting the findings of this study are presented in the article. The raw data are available on request.
